# Judicialization of Zolgensma in the Ministry of Health: costs and clinical profile of patients

**DOI:** 10.11606/s1518-8787.2024058005899

**Published:** 2024-07-24

**Authors:** Ana Katheryne Miranda Kretzschmar, Ellen Teixeira, Dayani Galato, Everton Nunes da Silva

**Affiliations:** I Universidade de Brasília Faculdade de Ceilândia Programa de Pós-Graduação em Ciências e Tecnologias da Saúde Brasília DF Brasil Universidade de Brasília. Faculdade de Ceilândia. Programa de Pós-Graduação em Ciências e Tecnologias da Saúde. Brasília, DF, Brasil; II Universidade de Brasília Faculdade de Ceilândia Brasília DF Brasil Universidade de Brasília. Faculdade de Ceilândia. Curso de Farmácia. Brasília, DF, Brasil; III Universidade de Brasília Faculdade de Ceilândia Brasília DF Brasil Universidade de Brasília. Faculdade de Ceilândia. Curso de Saúde Coletiva. Brasília, DF, Brasil

**Keywords:** Spinal Muscular Atrophy, Onasemnogene Abeparvovec, Health Judicialization, Cost Analysis

## Abstract

**OBJECTIVE:**

To investigate the costs and profile of patients who have filed a lawsuit against the Ministry of Health for the treatment of spinal muscular atrophy (SMA) with the onasemnogene abeparvovec (Zolgensma^®^).

**METHODS:**

This is a cross-sectional, descriptive study with a census design, based on records of lawsuits filed against the Ministry of Health between January 2019 and September 2022. Data was requested from the Ministry of Health via the Access to Information Act. Information was extracted on the epidemiological profile of the beneficiaries of the lawsuits, as well as the expenses spent by the Ministry of Health in cases where the requests were granted.

**RESULTS:**

136 lawsuits were identified, of which 113 (83%) were favorable to patients at a cost of R$ 944.8 million in the period analyzed. Demographic (gender and age), clinical (SMA subtypes, use of ventilatory or nutritional support), and lawsuit (type of legal service) characteristics were not associated with the granting of lawsuits. Prior use of medication (nusinersena or ridisplam) was associated with the dismissal of lawsuits. Of the 113 lawsuits granted in favor of patients, only six (5.3%) would meet the criteria currently established by the National Committee for Health Technology Incorporation - Conitec (children up to six months without ventilatory and nutritional support). R$ 146 million was spent on supplying Zolgensma to children over the age of two, which is outside the recommendation contained in the drug’s package leaflet.

**CONCLUSIONS:**

The Ministry of Health incurs a high cost with the judicialization of Zolgensma for SMA, representing 2.45% of total spending on medicines in the Unified Health System, including spending by the three administrative spheres. Some of the lawsuits have been granted in disagreement with the criteria established by health technology assessment agencies and the drug manufacturer’s recommendations.

## INTRODUCTION

Spinal muscular atrophy (SMA) 5q is a rare disease of autosomal recessive genetic inheritance that causes progressive degeneration of the spinal motor neuron. The main symptoms of this disease include progressive loss of movement, muscle weakness, paralysis, and respiratory failure^1^. In SMA, levels of the motor neuron survival protein (SMN) are reduced due to alterations in the genes that code for it: SMN1 and SMN2. Only mutations in the SMN1 gene that cause SMA and the presence of multiple copies of the SMN2 gene correlate with the clinical severity of SMA, as the greater the number of copies of the SMN2 gene, the slower the progression of signs and symptoms^2^. SMA 5q can be classified into five subtypes: 0, I, II, III, and IV, which differ in age and onset of symptoms. Type 0 is the most severe form; it starts in the prenatal period and babies generally don’t survive beyond six months. Type I manifests before the age of six months, with children generally unable to sit up on their own and requiring nutritional and ventilatory support. Type II manifests between seven and 18 months, children sit with support and keep their bodies in balance, and breathing and feeding difficulties appear to a lesser degree. Type III appears after 18 months and, depending on the progression of the disease, children can walk independently. Type IV represents 5% of cases, manifesting in adulthood and there is no complete loss of ability^3,4^. Although this is not a determining correlation, generally most patients with SMA type I have two copies of the SMN2 gene and patients with SMA types II and III can have three or four copies^5^.

There is no cure for SMA and there are currently three drugs available for treatment, all registered by the National Health Surveillance Agency (Anvisa): Spinraza^®^ (nusinersena), Everysdi^®^ (risdiplam), and Zolgensma^®^ (onasemnogene abeparvovec)^6^. The Ministry of Health’s National Committee for Health Technology Incorporation (Conitec) recommended that these drugs be incorporated into the Unified Health System (SUS). In all its decisions, Conitec evaluated the published studies and established certain criteria for making the drugs available free of charge. The standardization of any medicine for supply in the public system considers technical-scientific analyses based on the best available evidence, including efficacy, safety, cost-effectiveness and budget impact^9^and there is no hierarchy between them. In other words, depending on the results and quality of the evidence, decision-makers may place more emphasis on one type of evidence than the others. For example, in oncology, budget impact and effectiveness were the most frequently used criteria in Conitec’s recommendations^14^.

Unlike other drugs, Zolgensma is a gene therapy and for a long time was considered the most expensive drug in the world. At the beginning of 2023, each vial cost an average of US$ 2.5 million^15^. The studies published for this drug consider a number of particularities, and there is discussion about the clinical relevance of the endpoints for assessing efficacy^16^. In SUS, Zolgensma was incorporated in December 2022 only for the treatment of children with SMA type I, up to six months old, who are off invasive ventilation for more than 16 hours a day. According to Conitec, the available evidence on efficacy and safety is for a population of up to six months old, with type I SMA, without the use of permanent invasive mechanical ventilation^13^.

Some international agencies and bodies have also evaluated the drug and, like Conitec, have also established criteria for its supply. The Scottish Medicines Consortium (SMC) approved the drug for reimbursement only for patients with SMA type I^17^. The Canadian Agency for Drugs and Technologies in Health (CADTH) and the UK’s National Institute for Health and Care Excellence (NICE) also limited the use of the drug to patients up to six months of age, with SMA type I, without the need for permanent feeding or ventilatory support^18,19^.

The cost of treating SMA with Zolgensma is prohibitive even for those on high incomes, and its regulation has become increasingly frequent in the SUS. Because it is a gene therapy, many families see it as a curative product^15^. In addition, it has only recently been offered by the SUS and, as recommended by Conitec, it only covers one type of SMA, with specific criteria to be met.

Judicial decisions must be made carefully, as they can generate risks to the management of public health actions and services, putting the collective dimension of health at risk^20^. Many studies have shown that the benefits of Zolgensma have been achieved by populations with a specific clinical profile. It is believed that most of the decisions in favor of the author are based solely on individual requests and granted without considering the public policies formulated and the published evidence^21^. No research to date has evaluated the patient profile and monetary impact of the judicialization of Zolgensma within the SUS.

The aim of this study was to investigate the profile of patients who have filed lawsuits against the Ministry of Health to obtain the drug Zolgensma for the treatment of SMA, as well as the costs incurred by the Ministry of Health in cases where the lawsuits are upheld. It also estimated the costs that could be avoided if the criteria established by Conitec were considered by the judiciary.

## METHODS

At the federal level, SUS is composed by the Ministry of Health, but actions are also decentralized to the states and municipalities, with each entity having its own co-responsibilities. The Ministry of Health is the national manager that draws up, regulates, supervises, monitors and evaluates health policies and actions^24^. The Department for the Management of Judicial Demands in Health (DJUD) is part of the structure of the Ministry of Health and is responsible for coordinating, supervising, proposing measures, and developing mechanisms for the management, control, and monitoring of processes relating to judicial demands for medicines, supplies, medical, and hospital material and the contracting of services for SUS users^25^.

### Study design

This is a cross-sectional, descriptive study with a census design, based on records of lawsuits filed against the Ministry of Health to obtain the drug Zolgensma for the treatment of SMA. The data was requested from the Ministry of Health and corresponds to all lawsuits filed between January 2019 and September 2022. Based on the data received, information was extracted on the epidemiological profile of the beneficiaries of the lawsuits, as well as the expenses spent by the Ministry of Health in cases where the requests were granted.

### Population

The study population was made up of individuals diagnosed with SMA and who were the beneficiaries of a lawsuit against the Ministry of Health to obtain the drug Zolgensma. All individuals were identified during the study period, corresponding to the census design for the period under investigation. The following were excluded: 1) patients with diseases other than SMA; 2) patients with SMA but who requested medicines other than Zolgensma; and 3) lawsuits filed in periods other than the study timeframe.

### Variables and Data Source

The data, which was requested from the Department of Judicial Demands in Health via the Access to Information Act, was provided in an anonymized form and did not contain information that could identify the patients, such as name and affiliation.

Information was obtained on: 1) patients’ demographic characteristics, including age (in months) and gender (male and female); 2) patients’ clinical characteristics, including SMA classification (subtype 0, I, II, III, and IV), use of ventilatory support (yes or no), use of nutritional support (yes or no); medication previously used (none, nusinersena, or ridisplam); 3) information on the lawsuit, such as type of patient representation (private or public legal service) and outcome of the lawsuit (granted or dismissed).

For the cases in which patients had their lawsuits granted, information was obtained on the amount spent by the Ministry of Health (in current values R$), including the cost of the drug and any additional procedures (hospitals, application, medical fees, and transportation). In addition, information was obtained on how the lawsuit was paid for (direct purchase of the drug or court deposit) and the patient’s age at the time of the lawsuit.

### Data Analysis

The data obtained in the study was analyzed descriptively, using measures of central tendency and dispersion for numerical variables and frequency measures such as absolute numbers and proportions for categorical variables. The chi-squared test was then carried out to identify possible associations between the exposure variables and the outcome of the lawsuit being granted, and those with a p-value less than or equal to 0.05 were considered significant.

To relate the patient profile to the available evidence, two cut-off points were considered: 1) information from the drug’s package leaflet, in which the drug Zolgensma is recommended for patients with SMA type I, or up to three copies of the SMN2 gene, aged up to two years old^8^; and 2) clinical recommendation reports drawn up by health technology assessment agencies (Brazilian and from other countries), restricting the use of Zolgensma to SMA patients up to six months of age and without the need for permanent feeding or ventilatory support, as is the case with Conitec, SMC, CADTH and NICE^13,17^. The criteria for recommending the use of the drug are set out in Chart 1.

**Chart 1:** Recommendations and restrictions on the use of Zolgensma by the manufacturer and health technology assessment agencies.

Evidence	Age	Type of SMA	Restrictions on use
Package leaflet (manufacturer Novartis)^8^	Under two years old	SMA type I or up to three copies of the SMN2 gene	The use of Zolgensma in patients with advanced SMA has not been evaluated, for example: total paralysis of the limbs and permanent dependence on ventilation.
Conitec^13^	Up to six months	AME5q type I	Children on invasive ventilation for more than 16 hours a day.
SMC^16^	Does not define age	SMA type I, or up to three copies of the SMN2 gene, in which patients are expected to develop SMA type I	No restrictions on use.
CADTH^17^	Up to six months	SMA patients who are symptomatic or pre-symptomatic with one to three copies of the SMN2 gene	Children who need permanent feeding or ventilatory support (invasive or non-invasive).
NICE^18^	Up to six months or age seven to 12 months, with treatment agreed by the multidisciplinary team	AME5q type I	Tracheostomized children and those on permanent ventilation for more than 16 hours a day. Use in children aged seven to 12 months is only indicated if the treatment will give them a 70% chance of sitting up independently.

Source: own elaboration, based on the references indicated in column 1.

SMA: Spinal muscular atrophy; Conitec: National Commission for the Incorporation of Technologies; SMC: Scottish Medicines Consortium; CADTH: Canadian Agency for Drugs and Technologies in Health; NICE: National Institute for Health and Care Excellence.

### Ethical aspects

The study was approved by the Research Ethics Committee of the Faculdade de Ceilândia of the Universidade de Brasília, under registration number CAAE 69455923.0.0000.8093. The free and informed consent form was waived as the data was provided by the Ministry of Health in an anonymized form, without identifying the participants by name.

## RESULTS

During the period analyzed, 136 lawsuits for the drug Zolgensma for the treatment of SMA were identified within the Ministry of Health, of which 113 (83%) were favorable to the beneficiaries of the lawsuits. Demographic (gender and age), clinical (SMA subtypes, use of ventilatory or nutritional support), and lawsuit (type of legal service) characteristics were not associated with the granting of lawsuits. Only the previous use of medication for SMA (nusinersena or ridisplam) was associated with the dismissal of lawsuits. SMA I was the most frequent subtype in the lawsuits, representing 118 lawsuits (86.7%) of the total identified in the period under investigation (Table 1).

**Table 1:** Information on legal claims for the drug Zolgensma within the Ministry of Health between January 2019 and September 2022, Brazil.

Characteristics	Court order granted^a^		Legal claim dismissed		p-value
	No. of applicants	%	No. of applicants	%	
Sex					0.590
Female	52	46	12	52.2	
Male	61	54	11	47.8	
Patient’s age on application					0.323
0-6 months	31	27.4	4	17.4	
7-12 months	33	29.2	9	39.1	
13-18 months	27	23.9	5	21.7	
19-24 months	19	16.8	3	13.0	
25 months or older	3	2.7	2	8.7	
Patient’s age at judicial compliance^a^					
0-6 months	10	9.2			
7-12 months	16	14.7			
13-18 months	33	30.3			
19-24 months	32	29.4			
25 months or older	18	16.5			
Type of SMA					0.438
I	99	87.6	19	82.6	
II	13	11.5	3	13	
III	1	0.9	1	4.3	
Type of representation					0.161
Private	110	97.3	21	91.3	
Public	3	2.7	2	8.7	
Use of other medicines^b^					0.025
Yes	45	39.8	15	65.2	
No	68	60.2	8	34.8	
Ventilatory support					0.701
Yes	54	47.8	12	52.2	
No	59	52.2	11	47.8	
Nutritional support					0.974
Yes	34	30.1	7	30.4	
No	79	69.9	16	69.6	

^a^ Of the 113 cases granted, four had not yet been complied with.

^b^ Medicines for the management of SMA: nusinersena or risdiplam.

When analyzing only the cases in which the lawsuits were granted, 97.3% of the patients were up to two years old when the lawsuit was filed, in line with the drug manufacturer’s package leaflet recommendations. However, this percentage drops to 84.5% when analyzing the patient’s age at the time of the lawsuit (payment of the medication). Considering the limit of up to six months of age for obtaining Zolgensma for the treatment of SMA established in a clinical protocol in Brazil by Conitec and other international agencies (CADTH and NICE), these percentages fall to 27.4% and 9.2%, respectively (Table 1).

It should be noted that of the 113 lawsuits that were granted, only 109 had information on the cost and age of compliance. Four lawsuits that had been granted had not yet been met with the delivery of the medication or a court deposit. During the study period, the Ministry of Health disbursed R$ 944.8 million to meet 109 lawsuits for the drug Zolgensma to treat SMA, with an average expenditure of R$ 8.67 million per patient. In 43 lawsuits, additional funds were requested for payment of medical fees, hospitalizations, and transport of patients to the place where the drug would be applied, totaling R$ 3.2 million in the period investigated (Table 2).

**Table 2:** Total expenditure by the Ministry of Health on legal claims for the drug Zolgensma by type of SMA from January 2019 to September 2022.

Type of SMA	Cost of medication		Additional costs		Total cost (R$)
	Quantity	Cost (R$)	Quantity	Cost (R$)	
I	99^a^	833,386,445.27	37	2,950,690.29	836,337,135.56
II	13^b^	99,377,369.67	6	271.240.14	99,648,609.81
III	1	8,812,225.00	0	R$ 0.00	8,812,225.00
Total cost					944,797,970.37

^a^ Three lawsuits had not yet been granted and therefore their costs were not considered.

^b^ One lawsuit had not yet been granted and therefore its costs were not considered.

Table 3 lists the costs of legalizing the drug Zolgensma with the criteria defined in clinical protocols in different countries, including Brazil. Of the 113 lawsuits granted in favor of patients, only six (5.3%) would meet the criteria established by Conitec (children up to six months without ventilatory and nutritional support). As a result, R$ 891 million spent on lawsuits would not meet the criteria established in Brazil’s clinical protocol. Another point worth highlighting is the R$ 146 million spent on supplying Zolgensma to children over the age of two, which is outside the recommendation contained in the drug’s package leaflet, established by the manufacturer (Table 3).

**Table 3:** Amount and expenditure incurred by the Ministry of Health to meet the legal demand for Zolgensma for the treatment of SMA, stratified by clinical criteria adopted by international health technology assessment agencies.

Group	SMA type I		SMA type II			SMA type III
	n	Cost (R$)	n	Cost (R$)	n	Cost (R$)
Children up to six months without ventilatory and nutritional support	6	53,782,491.83	0	0.00	0	0.00
Children up to six months with ventilatory and nutritional support	4	36,731,461.25	0	0.00	0	0.00
Children > six months and ≤ 24 months with ventilatory and nutritional support	42	384,831,441.21	0	0.00	0	0.00
Children > six months and ≤ 24 months without ventilatory and nutritional support	30	247,183,951.57	9	76,249,872.02	0	0.00
Children > 24 months	17^a^	113,807,789.70	4^b^	23,398,737.79	1	8,812,225.00
Total	99	836,337,135.56	13	99,648,609.81	1	8,812,225.00

^a^ Three lawsuits had not yet been granted and therefore their costs were not considered.

^b^ One lawsuit had not yet been granted and therefore its costs were not considered.

## DISCUSSION

During the period analyzed, the Ministry of Health disbursed R$ 944.8 million to comply with lawsuits over the drug Zolgensma for the treatment of SMA. The annual average was R$ 251.9 million for the care of approximately 29 patients, making it the largest expenditure on judicialization at the federal level. Excluding spending on Zolgensma for the treatment of SMA, the Ministry of Health spent R$ 802.6 million on other lawsuits in 2019, serving more than 3,000 beneficiaries^26^. The comparison becomes more disparate when considering the total spending on medicines in the SUS, which totaled R$ 10.29 billion in the same year^27^including the treatment of all diseases and health problems in the country through health policies, which, in theory, represents the treatment of more than 200 million inhabitants. The cost of regulating Zolgensma for SMA would represent 2.45% of total spending on medicines in the SUS, including spending by the three administrative spheres (Ministry of Health, states, and municipalities). It should also be noted that, of the total expenditure on the regulation of Zolgensma for SMA (R$ 944.8 million), only R$ 53.8 million (5.7%) would meet the criteria established by Conitec and R$ 798.7 million (84.5%) in relation to the drug manufacturer’s package leaflet. However, considering that the package leaflet itself does not guarantee efficacy and safety in patients with total paralysis of the limbs and permanent dependence on ventilation, this expense may be higher, as it is not known whether the use of ventilatory support reported was permanent.

The amount disbursed also varied in terms of the form of payment. Out of 109 cases, in only 18 of them was the medicine purchased directly by the Ministry of Health, with the unit value of the kit being R$ 5,722,712.79. For the cases in which there was a deposit for direct purchase by the beneficiary or person responsible for the action, the average value of the medicine was R$ 9,215,849.51 (ranging from R$ 4,179,008.39 to R$ 12,105,487.50). In 23 deposit processes, the drug kit exceeded R$ 11,000,000.00, reaching R$ 12,105,487.50. The amount paid via deposit is higher than the amount regulated by the Drug Market Regulation Chamber (CMED), set at R$ 6.5 million^15^.

The types of SMA in this study are similar to the data published on the disease. Most of the cases granted correspond to patients with type I SMA, precisely because it is the most severe subtype^28^which requires greater care and has a higher prevalence in Brazil^29^.

The absence of prior drug treatment contributed to the decision to grant Zolgensma in court cases in the context of the Ministry of Health. This pattern is in line with the available evidence, since exposure to previous treatments was one of the exclusion criteria in the clinical trials^30,31^. The same did not occur in terms of age at the start of treatment (up to six months) and the use of ventilatory and nutritional support. The clinical trials available only included patients aged up to six months and carrying two copies of the SMN2 gene; exclusion criteria included the use of invasive ventilatory support (tracheotomy) or non-invasive ventilatory support for an average of ≥ 6 hours a day, as well as patients who had swallowing problems (signs of aspiration/inability to tolerate liquid)^32^. The children’s age is a very relevant factor for treatment, as once motor damage has set in, there is no way of reversing it^35^.

In *Clinical Trials* there are 13 studies of the drug Zolgensma for the treatment of SMA (search made on October 1^st^, 2023). However, only five have published results (NCT03837184, NCT03306277, NCT03461289, NCT03505099, NCT03381729). In all the studies with results, the children were aged six months or younger, had up to two copies of the SMN2 gene, were not on invasive or non-invasive ventilatory support for more than ≥6 hours a day, and were on nutritional support^30,31,33,34,36^. In most of the court cases that were granted, 72.6% of the children were older than six months when considering the date of the petition. It is also worth mentioning that the drug’s package insert indicates treatment for pediatric patients up to two years of age^8^. In total, 16.5% of the children took the drug over the age of two.

Other international agencies have also restricted the use of Zolgensma for the treatment of SMA to certain clinical conditions and age at the start of treatment. The Canadian agency and NICE in England have limited the use of the drug to patients up to six months of age, with SMA type I, without the need for feeding support or ventilatory support (invasive or non-invasive)^17,18^. These recommendations are similar to those adopted in Brazil, recommended by Conitec. In Scotland, the drug has been approved for reimbursement only for patients under one year old who are in the pre-symptomatic phase, i.e. those who have not yet shown symptoms of the disease^17^. The Institute for Clinical and Economical Review (ICER) evaluated the drug and compared its cost-effectiveness with nusinersena, concluding that Zolgensma is more cost-effective if applied in the pre-symptomatic phase of patients without symptoms or younger than two/three months^37^.

The large number of lawsuits to obtain Zolgensma outside the parameters indicated by Conitec (age up to six months and absence of ventilatory and nutritional support)^13^ may be related to the difficulty in obtaining an early diagnosis of SMA in Brazil. As it is a disorder with a low incidence and which progresses progressively, diagnosing SMA can become a challenge, as signs of SMA can be confused with other neuropathologies^38^. In addition, there are difficulties in knowing the clinical signs, a lack of specialists and the absence of genetic testing to confirm the diagnosis. The survey “Portrait of SMA in Brazil” showed that only 1% of patients had been diagnosed before birth and 11% up to one year old. In addition, around 33% of individuals report that the diagnosis was only possible after consulting five doctors or more^39^. A study of groups of patients from Europe, Australia, the United States, and Asia compared the age at which the first symptoms of SMA appeared and the age at which the diagnosis was confirmed. The results showed that delays in diagnosis can take months or years^40^.

Even with the benefits presented by the efficacy studies of the drug Zolgensma for the population up to six months, without some defined comorbidities (such as the use of ventilatory support), there is still a discussion about the clinical relevance of the published outcomes. So far, the studies show that the motor milestone achieved by the patients was reaching an independent position for 30 seconds at 18 months old. In fact, some milestones have been redefined over the course of the studies, which reduces the reliability of the data analysis^16^. As this is a new treatment, many studies are still underway and there is no published data on the real benefits of long-term therapy.

### Strengths and limitations of the study

This study contributed by summarizing administrative data on lawsuits against the Ministry of Health to obtain the drug Zolgensma for SMA. The census design at the federal level made it possible to identify the costs and the convergence of judicial approval with the clinical criteria established by international health technology assessment agencies, including Conitec.

Some limitations of the study need to be mentioned. Firstly, a large part of the period analyzed took place during the Covid-19 pandemic, which may have reduced demand for petitions relating to SMA and increased demands relating to Covid-19. Secondly, the absence of more detailed information on the use of ventilatory support in medical documents. It is believed that the number of children on ventilatory support may be higher because, considering the delay in complying with the court order, many children may have needed more care due to the progression of the disease. Only a more detailed study of this cohort will be able to ascertain the profile of the patients during the application of the drug and provide answers as to the benefits of treatment with Zolgensma. Finally, it should be noted that this study did not focus on assessing whether the criteria used by health technology assessment agencies, including Conitec, for incorporating Zolgensma into health systems are correct. Future research in this direction is therefore recommended.

### Implications of the results for health policies

This study corroborates the importance of early diagnosis of SMA and, consequently, its coverage in the SUS, given that more than half of the children who had the drug legalized were outside the parameters indicated by Conitec. In addition, the drug was used in children with a different profile to that indicated by the evidence in the literature (published studies and the manufacturer’s own package leaflet) and the recommendation protocols drawn up by health technology assessment agencies.

As it is a health condition with a low incidence and which progresses progressively, the diagnosis of SMA can become a challenge and take months or years, due to the similarity of the initial symptoms with other neuropathologies, the lack of specialists and the absence of a genetic test to confirm the diagnosis. These factors can affect, for example, access to the drug by the SUS, according to the criteria recommended by Conitec. Public health policies need to encourage early diagnosis of SMA.

Also noteworthy is the importance of the Technical Support Centers for the Judiciary (NAT-JUS) in preparing opinions based on evidence-based medicine and the National Council of Justice’s (CNJ) Health Forum in monitoring health demands. Case law needs this technical support in order to carry out a thorough analysis of the specific case, and it is essential to adopt evidence-based medicine for decision-making. Rising health costs, the recognition of limited resources, the need to guarantee constitutional rights, and the growing intervention of the Judiciary make Health technology assessment (HTA) an essential part of the decision-making process.

The discussion on how to comply with the lawsuit should be broadened, given that direct payment by the Ministry of Health resulted in lower costs for the acquisition of the medicine compared to the judicial deposit. A saving of R$ 332 million would have been achieved in the period investigated if the 95 lawsuits that were complied with by means of a judicial deposit were made by means of direct payment.

Due to uncertainties about the long-term effects of the drug and concerns about the sustainability of the SUS budget, the importance of implementing risk-sharing agreements for high-cost drugs used in rare diseases is highlighted, even for the judicial scenario. It is important for the judiciary to have an adequate technical vision when analyzing claims and to avoid making decisions that go against the health system and science.

## References

[1] Mendonça RH, Matsui C, Polido GJ, Silva AM, Kulikowski L, Torchio Dias A (2020). Intragenic variants in the SMN1 gene determine the clinical phenotype in 5q spinal muscular atrophy. Neurol Genet.

[2] Prior TW, Leach ME, Finanger E, Adam MP, Mirzaa GM, Pagon RA (2020). Spinal muscular atrophy.

[3] Costa JO, Costa CCP, Lima NS (2021). Genes de sobrevivência do neurônio motor e a atrofia muscular espinhal 5q. Genética Escola.

[4] Pera MC, Coratti G, Berti B, D'Amico A, Sframeli M, Albamonte E (2020). Diagnostic journey in Spinal Muscular Atrophy: is it still an odyssey?. PLoS One.

[5] Araujo APQC, Giuliani A, Bonfim D, Loriato D, Zanotelli E, Braga F (2019). Guia de discussão sobre Atrofia Muscular Espinhal no Brasil: Trabalhando hoje para mudar o amanhã.

[6] Agência Nacional de Vigilância Sanitária (2017). Anvisa concede registro ao medicamento Spinraza.

[7] Agência Nacional de Vigilância Sanitária (2020). EVRYSDI ® (risdiplam): novo registro.

[8] Agência Nacional de Vigilância Sanitária (2020). Aprovado registro de produto de terapia gênica.

[9] Ministério da Saúde (BR), Comissão Nacional de Incorporação de Tecnologias no Sistema Único de Saúde (2019). Relatório de Recomendação N o 449 - Nusinersena para atrofia muscular espinhal 5q.

[10] Ministério da Saúde (BR), Comissão Nacional de Incorporação de Tecnologias no Sistema Único de Saúde (2021). Relatório de Recomendação N o 595 - Nusinersena para tratamento da Atrofia Muscular Espinhal 5q tipo II e III (início tardio).

[11] Ministério da Saúde (BR), Comissão Nacional de Incorporação de Tecnologias no Sistema Único de Saúde (2022). Relatório de Recomendação N o 710 - Risdiplam para o tratamento de atrofia muscular espinhal (AME) tipo II e III.

[12] Ministério da Saúde (BR), Comissão Nacional de Incorporação de Tecnologias no Sistema Único de Saúde (2022). Relatório de Recomendação N o 709 - Risdiplam para o tratamento de atrofia muscular espinhal (AME) tipo I.

[13] Ministério da Saúde (BR), Comissão Nacional de Incorporação de Tecnologias no Sistema Único de Saúde (2022). Relatório de Recomendação N o 793 - Onasemnogeno abeparvoveque para o tratamento de atrofia muscular espinhal (AME).

[14] Campolina AG, Yuba TY, Soárez PC (2022). Decision criteria for resource allocation: an analysis of CONITEC oncology reports. Cien Saude Colet.

[15] Guimarães R (2023). Novos desafios na avaliação de tecnologias em saúde (ATS): o caso Zolgensma. Cien Saude Colet.

[16] Riera R, Bagattini ÂM, Pachito D (2019). Eficácia, segurança e aspectos regulatórios dos medicamentos órfãos para doenças raras: o caso Zolgensma ®. Cad Iberoam Direito Sanit.

[17] Scottish Medicines Consortium (2021). Decision explained - Medicine: onasemnogene abeparvovec (brand name: Zolgensma ® ).

[18] Canadian Agency for Drugs and Technologies in Health (2021). CADTH Common drug review: onasemnogene abeparvovec.

[19] National Institute for Health and Care Excellence (2021). Onasemnogene abeparvovec for treating spinal muscular atrophy: highly specialised technologies guidance.

[20] Chieffi AL, Barata RC (2010). Ações judiciais: estratégia da indústria farmacêutica para introdução de novos medicamentos. Rev Saude Publica.

[21] Araújo LC, Moita MP, Silva LCS, Mesquita KO, Vasconcelo FJL, Dias MS (2021). A Judicialização do acesso a medicamentos no Brasil: revisão integrativa da literatura. Sanare.

[22] Oliveira YM, Braga BS, Pereira SP, Ferreira MA, Braga BSF, Pereira SPD (2020). Judicialização de medicamentos: efetivação de direitos ou ruptura das políticas públicas?. Rev Saude Publica.

[23] Vieira FS (2023). Judicialização e direito à saúde no Brasil: uma trajetória de encontros e desencontros. Rev Saude Publica.

[24] Ferreira GA, Ferreira CA (2022). O Sistema Único de Saúde (SUS) brasileiro: trajetória e perspectivas. Rev Direito Debate.

[25] Brasil (2023). Decreto N o 11.391, de 20 de janeiro de 2023. Altera o Decreto n o 11.358, de 1 o de janeiro de 2023, que aprova a Estrutura Regimental e o Quadro Demonstrativo dos Cargos em Comissão e das Funções de Confiança do Ministério da Saúde. Diário Oficial União.

[26] Costa CA (2022). Gastos com a judicialização de tecnologias de saúde: um estudo empírico no executivo federal do Brasil.

[27] Ministério da Saúde (BR), Fundação Oswaldo Cruz (2022). Contas de saúde na perspectiva da contabilidade internacional: conta SHA para o Brasil, 2015 a 2019.

[28] Silva FS, Rodrigues JMP, Brito RN, Macedo TC, Borgmann AD (2021). Intervenção fisioterapêutica na atrofia muscular espinhal: revisão de literatura. Rev Neurocienc.

[29] Verhaart IE, Robertson A, Wilson IJ, Aartsma-Rus A, Cameron S, Jones CC (2017). Prevalence, incidence and carrier frequency of 5q-linked spinal muscular atrophy - a literature review. Orphanet J Rare Dis.

[30] Strauss KA, Farrar MA, Muntoni F, Saito K, Mendell JR, Servais L (2022). Onasemnogene abeparvovec for presymptomatic infants with two copies of SMN2 at risk for spinal muscular atrophy type 1: the Phase III SPR1NT trial. Nat Med.

[31] Novartis (2017). Study of AVXS-101 for Sitting But Non-ambulatory Patients With Spinal Muscular Atrophy (NCT03381729).

[32] Novartis (2017). Gene transfer clinical trial for spinal muscular atrophy type 1.

[33] Day JW, Finkel RS, Chiriboga CA, Connolly AM, Crawford TO, Darras BT (2021). Onasemnogene abeparvovec gene therapy for symptomatic infantile-onset spinal muscular atrophy in patients with two copies of SMN2 (STR1VE): an open-label, single-arm, multicentre, phase 3 trial. Lancet Neurol.

[34] Mercuri E, Muntoni F, Baranello G, Masson R, Boespflug-Tanguy O, Bruno C (2021). Onasemnogene abeparvovec gene therapy for symptomatic infantile-onset spinal muscular atrophy type 1 (STR1VE-EU): an open-label, single-arm, multicentre, phase 3 trial. Lancet Neurol.

[35] Sales CMP, Soliani FCBG, Sanches ACB (2022). Farmacoterapia da atrofia muscular espinhal Pharmacotherapy of spinal muscular atrophy. J Health Sci Inst.

[36] Novartis Gene Therapies (2019). Single-dose gene replacement therapy clinical trial for patients with spinal muscular atrophy type 1 with one or two SMN2 Copies Delivering AVXS-101 by Intravenous Infusion (NCT03837184).

[37] Institute for Clinical and Economic Review (2019). Spinraza ® and Zolgensma ® for spinal muscular atrophy: effectiveness and value: final evidence report.

[38] Baioni MT, Ambiel CR (2010). Spinal muscular atrophy: diagnosis, treatment and future prospects. J Pediatr (Rio J).

[39] Roche IN (2022). Retrato da AME no Brasil: um mapeamento com 144 respondentes revela o cenário desafiador de pessoas com atrofia muscular espinhal e seus cuidadores, do diagnóstico à assistência. E também aponta caminhos para superar essas barreiras.

[40] Lin CW, Kalb SJ, Yeh WS (2015). Delay in diagnosis of spinal muscular atrophy: a systematic literature review. Pediatr Neurol.

