# Supplementary material for: Judicialization of Zolgensma in the Ministry of Health: costs and clinical profile of patients
Source: Rev Saude Publica. 2024 Jul 24;58:36. doi: 10.11606/s1518-8787.2024058005899 (PMC11319041; doi:10.11606/s1518-8787.2024058005899)
Supplement: Translation

# Translation: Judicialização do Zolgensma no Ministério da Saúde: custos e perfil clínico dos pacientes

**Authors:** Ana Katheryne Miranda Kretzschmar, Ellen Teixeira, Dayani Galato, Everton Nunes da Silva

**Affiliations:** I Brasil Universidade de Brasília. Faculdade de Ceilândia. Programa de Pós-Graduação em Ciências e Tecnologias da Saúde. Brasília, DF, Brasil; II Brasil Universidade de Brasília. Faculdade de Ceilândia. Curso de Farmácia. Brasília, DF, Brasil; III Brasil Universidade de Brasília. Faculdade de Ceilândia. Curso de Saúde Coletiva. Brasília, DF, Brasil

**Keywords:** Atrofia Muscular Espinal, Onasemnogene Abeparvoveque, Judicialização da Saúde, Custos e Análise de Custo

## Abstract

**OBJETIVO:**

Investigar os custos e o perfil dos pacientes que demandaram judicialmente o onasemnogene abeparvoveque (Zolgensma^®^) para o tratamento da atrofia muscular espinhal (AME) no Ministério da Saúde.

**MÉTODOS:**

Estudo transversal, de natureza descritiva e desenho censitário, com base em registros de ações judiciais movidas contra o Ministério da Saúde no período de janeiro de 2019 a setembro de 2022. Os dados foram solicitados ao Ministério da Saúde, via Lei de Acesso à Informação. Foram extraídas informações sobre o perfil epidemiológico dos beneficiários das ações judiciais, bem como os gastos dispendidos pelo Ministério da Saúde nos casos de deferimento das solicitações.

**RESULTADOS:**

Foram identificados 136 processos judiciais, dos quais 113 (83%) foram favoráveis aos pacientes ao custo de R$ 944,8 milhões no período analisado. Características demográficas (sexo e idade), clínicas (subtipos da AME, uso de suporte ventilatório ou nutricional) e do processo judicial (tipo de serviço advocatício) não foram associadas com o deferimento das ações judiciais. O uso prévio de medicamento (nusinersena ou ridisplam) foi associado com o indeferimento dos processos judiciais. Das 113 ações judiciais concedidas em favor dos pacientes, apenas seis (5,3%) atenderiam aos critérios estabelecidos atualmente pela Comissão Nacional de Incorporação de Tecnologias – Conitec (crianças com até seis meses sem suporte ventilatório e nutricional). Houve dispêndio de R$ 146 milhões com o fornecimento do Zolgensma para crianças com idade superior a dois anos, que está fora da recomendação contida na bula do medicamento.

**CONCLUSÕES:**

O Ministério da Saúde incorre em um alto custo com a judicialização do Zolgensma para AME, representando 2,45% do gasto total com medicamentos no Sistema Único de Saúde, incluindo gastos das três esferas administrativas. Parte das demandas judiciais tem sido deferida em divergência com os critérios estabelecidos por agências de avaliação de tecnologias em saúde e recomendações do fabricante do medicamento.

## INTRODUÇÃO

A atrofia muscular espinhal (AME) 5q é uma doença rara, de herança genética autossômica recessiva, que provoca a degeneração progressiva do neurônio motor espinhal. Os principais sintomas desta doença incluem: perda progressiva dos movimentos, fraqueza muscular, paralisia e insuficiência respiratória^1^. Na AME, os níveis da proteína de sobrevivência do neurônio motor (SMN) encontram-se reduzidos, devido a alterações nos genes que a codificam: SMN1 e SMN2. Apenas as mutações no gene SMN1 que ocasionam a AME e a presença de múltiplas cópias do gene SMN2 se correlacionam com a gravidade clínica da AME, pois quanto maior o número de cópias do gene SMN2, menor é a velocidade de progressão dos sinais e sintomas^2^. A AME 5q pode ser classificada em cinco subtipos: 0, I, II, III e IV, que diferenciam entre si pela idade e início dos sintomas. O tipo 0 é a forma mais grave, tem início no pré-natal e geralmente os bebês não sobrevivem além dos seis meses de vida. O tipo I se manifesta antes dos seis meses de idade, sendo que geralmente as crianças não se sentam sozinhas e necessitam de suporte nutricional e ventilatório. O tipo II manifesta-se entre sete e 18 meses de idade, as crianças sentam-se com apoio e mantêm o corpo em equilíbrio, e as dificuldades respiratórias e de alimentação aparecem em menor nível. O tipo III aparece após os 18 meses de idade e, a depender da progressão da doença, as crianças podem andar de forma independente. O tipo IV representa 5% dos casos, manifestando-se na idade adulta e não há perda de habilidade por completo^3,4^. Apesar de não ser uma correlação determinante, geralmente a maior parte dos portadores de AME tipo I apresenta duas cópias do gene SMN2 e pacientes com AME tipo II e III podem ter três cópias ou quatro cópias^5^.

Não existe cura para a AME e atualmente existem três fármacos disponíveis para tratamento, todos registrados pela Agência Nacional de Vigilância Sanitária (Anvisa): Spinraza^®^ (nusinersena), Everysdi^®^ (risdiplam) e Zolgensma^®^ (onasemnogene abeparvoveque)^6^. A Comissão Nacional de Incorporação de Tecnologias (Conitec) do Ministério da Saúde, recomendou a incorporação desses medicamentos no Sistema Único de Saúde (SUS). Em todas as decisões, a Conitec avaliou os estudos publicados e estabeleceu alguns critérios para a disponibilização gratuita dos medicamentos. A padronização de qualquer medicamento para fornecimento no sistema público leva em consideração análises técnico-científicas a partir das melhores evidências disponíveis, incluindo eficácia, segurança, custo-efetividade e impacto orçamentário^9^, não havendo uma hierarquia entre elas. Ou seja, dependendo dos resultados e da qualidade das evidências, os tomadores de decisão podem dar mais ênfase por um tipo delas do que os demais. Por exemplo, em oncologia, o impacto financeiro e a efetividade foram os critérios mais frequentemente usados nas recomendações da Conitec^14^.

Diferentemente dos demais medicamentos, o Zolgensma é uma terapia gênica e por muito tempo foi considerado o medicamento mais caro do mundo. Cada frasco custava, no início de 2023, em média US$ 2,5 milhões^15^. Os estudos publicados para este medicamento levam em consideração uma série de particularidades, havendo uma discussão sobre a relevância clínica dos desfechos para avalição da eficácia^16^. No SUS, o Zolgensma foi incorporado em dezembro de 2022 somente para o tratamento de crianças com AME do tipo I, com até seis meses de idade, que estejam fora de ventilação invasiva acima de 16 horas por dia. De acordo a Conitec, as evidências disponíveis sobre eficácia e segurança são para uma população de até seis meses, com AME tipo I, sem uso de ventilação mecânica invasiva permanente^13^.

Algumas agências e órgãos internacionais também avaliaram o medicamento e, assim como a Conitec, também estabeleceram critérios para o seu fornecimento. A agência escocesa *Scottish Medicines Consortium* (SMC) aprovou o medicamento para reembolso apenas para pacientes com AME tipo I^17^. A agência canadense *Canadian Agency for Drugs and Technologies in Health* (CADTH) e a do Reino Unido *National Institute for Health and Care Excellence* (NICE) também limitaram o uso do medicamento a pacientes de até seis meses de idade, com AME tipo I, sem necessidade de alimentação permanente ou suporte ventilatório^18,19^.

O custo do tratamento da AME com Zolgensma torna-se proibitivo até mesmo para aqueles que possuem renda alta, e a sua judicialização tem se tornado cada vez mais frequente no SUS. Por se tratar de uma terapia gênica, muitas famílias o enxergam como um produto curativo^15^. Além disso, a sua oferta pelo SUS é recente e, conforme recomendação da Conitec, abrange apenas um tipo de AME, com critérios específicos a serem cumpridos.

As decisões judiciais proferidas devem ser realizadas de forma criteriosa, pois são capazes de gerar riscos ao gerenciamento das ações e serviços públicos de saúde, colocando em risco a dimensão coletiva da saúde^20^. Muitos estudos demonstram que os benefícios do Zolgensma foram atingidos por populações com perfil clínico específico. Acredita-se que grande parte das decisões favoráveis ao autor são baseadas apenas nas solicitações individuais e concedidas sem consideração das políticas públicas formuladas e das evidências publicadas^21^. Nenhuma pesquisa até o momento avaliou o perfil de pacientes e o impacto monetário da judicialização do Zolgensma no âmbito do SUS.

Este estudo tem como objetivo investigar o perfil dos pacientes que moveram processos judiciais contra o Ministério da Saúde para a obtenção do medicamento Zolgensma para o tratamento da AME, bem como os custos incorridos pelo Ministério da Saúde nos casos de deferimento das ações judiciais. Também se estimou os custos que poderiam ser evitados caso os critérios estabelecidos pela Conitec fossem considerados pelo Judiciário.

## MÉTODOS

O SUS é composto, em nível federal, pelo Ministério da Saúde, mas também há descentralização em ações nos estados e municípios, sendo que cada ente tem suas corresponsabilidades. O Ministério da Saúde é o gestor nacional que elabora, normatiza, fiscaliza, monitora e avalia as políticas e ações em saúde^24^. O Departamento de Gestão das Demandas em Judicialização na Saúde (DJUD) compõe a estrutura do Ministério da Saúde, e é responsável por coordenar, supervisionar, propor medidas e desenvolver mecanismos de gestão, controle e monitoramento dos processos referentes a demandas judiciais de medicamentos, insumos, material médico-hospitalar e a contratação de serviços destinados aos usuários do SUS^25^.

### Desenho do Estudo

Trata-se de estudo transversal, de natureza descritiva e desenho censitário, com base em registros de ações judiciais movidas contra o Ministério da Saúde para a obtenção do medicamento Zolgensma para tratamento da AME. Os dados foram solicitados ao Ministério da Saúde e correspondem a todas as ações judiciais registradas no período de janeiro de 2019 a setembro de 2022. Com base nos dados recebidos, foram extraídas informações sobre o perfil epidemiológico dos beneficiários das ações judiciais, bem como os gastos dispendidos pelo Ministério da Saúde nos casos de deferimento das solicitações.

### População

A população do estudo foram indivíduos com diagnóstico de AME beneficiários de ação judicial contra o Ministério da Saúde para obtenção do medicamento Zolgensma. Foram identificados todos os indivíduos no período do estudo, correspondendo ao desenho censitário no período sob investigação. Foram excluídos: 1) os pacientes com outras doenças, que não a AME; 2) pacientes com AME, mas que demandaram outros medicamentos, que não o Zolgensma; e 3) ações judiciais impetradas em períodos distintos ao horizonte temporal do estudo.

### Variáveis e Fonte de Dados

Os dados, solicitados ao Departamento de Demandas Judiciais em Saúde via Lei de Acesso à Informação, foram concedidos de forma anonimizada, não contendo informações que pudessem identificar os pacientes, como nome e filiação.

Foram obtidos informações sobre: 1) características demográficas dos pacientes, incluindo idade (em meses) e sexo (masculino e feminino); 2) características clínicas dos pacientes, contemplando a classificação da AME (subtipo 0, I, II, III e IV), uso de suporte ventilatório (sim e não), uso de suporte nutricional (sim ou não); medicamento utilizado previamente (nenhum, nusinersena ou ridisplam); 3) informações do processo judicial, como tipo de representação do paciente (serviço advocatício privado ou público) e resultado da ação judicial (deferido ou indeferido).

Para os casos em que houve ganho de causa para os pacientes, foram obtidas informações sobre o valor gasto pelo Ministério da Saúde (em valores correntes R$), incluindo custo com o medicamento e eventuais procedimentos adicionais (hospitais, aplicação, honorários médicos e transporte). Adicionalmente, foram obtidas informações sobre a forma de pagamento da ação judicial (compra direta do medicamento ou depósito judicial) e a idade do paciente no momento do cumprimento judicial.

### Análise dos Dados

Os dados obtidos no estudo foram analisados de forma descritiva, adotando-se medidas de tendência central e de dispersão para as variáveis numérica e medidas de frequência como números absolutos e proporções para as variáveis categóricas. Foi realizado posteriormente o teste do qui-quadrado para identificar possíveis associações entre as variáveis de exposição e o desfecho de deferimento da ação judicial, sendo consideradas significativas aquelas com p-valor menor ou igual a 0,05.

Para relacionar o perfil dos pacientes com as evidências disponíveis, foram considerados dois pontos de corte: 1) informações da bula do medicamento, na qual o medicamento Zolgensma é recomendado para pacientes com AME tipo I, ou até três cópias do gene SMN2, com idade de até dois anos^8^ e 2) relatórios clínicos de recomendação elaborados por agências de avaliação de tecnologias em saúde (brasileira e de outros países), restringindo o uso do Zolgensma a pacientes com AME com até seis meses de idade e sem necessidade de alimentação permanente ou suporte ventilatório, como é o caso da Conitec, SMC, CADTH e NICE^13,17^. Os critérios de recomendação de utilização do medicamento estão discorridos no Quadro 1.

**Quadro 1:** Recomendações e restrições de utilização do Zolgensma pelo fabricante e pelas agências de avaliação de tecnologias em saúde.

Evidência	Idade	Tipo de AME	Restrições de uso
Bula do medicamento (fabricante Novartis)^8^	Abaixo de dois anos	AME tipo I ou até três cópias do gene SMN2	O uso de Zolgensma em pacientes com AME avançada não foi avaliado, por exemplo: paralisia total dos membros e dependência permanente de ventilação.
Conitec^13^	Até seis meses	AME5q tipo I	Crianças em ventilação invasiva acima de 16 horas por dia.
SMC^16^	Não define idade	AME tipo I, ou até três cópias do gene SMN2, em que se espera que os pacientes desenvolvam AME tipo I	Não informa restrições de uso.
CADTH^17^	Até seis meses	Pacientes com AME que são sintomáticos ou pré- sintomáticos com uma a três cópias do gene SMN2	Crianças que necessitam de alimentação permanente ou suporte ventilatório (invasivo ou não invasivo).
NICE^18^	Até seis meses ou idade de sete a 12 meses, com tratamento acordado pela equipe multidisciplinar	AME5q tipo I	Crianças traqueostomizadas e em ventilação permanente acima de 16 horas por dia. O uso em crianças de sete a 12 meses é indicado somente se o tratamento lhes dará 70% de chance de se sentar de forma independente.

Fonte: elaboração própria, com base nas referências indicadas na coluna 1.

AME: Atrofia muscular espinhal; Conitec: Comissão Nacional de Incorporação de Tecnologias; SMC: *Scottish Medicines Consortium*; CADTH: *Canadian Agency for Drugs and Technologies in Health*; NICE: *National Institute for Health and Care Excellence*.

### Aspectos Éticos

O estudo foi aprovado pelo Comitê de Ética em Pesquisa da Faculdade de Ceilândia da Universidade de Brasília, sob registro n° CAAE 69455923.0.0000.8093. Houve dispensa da aplicação do termo de consentimento livre e esclarecido, posto que os dados foram fornecidos pelo Ministério da Saúde de forma anonimizada, sem identificação nominal dos participantes.

## RESULTADOS

Durante o período analisado, foram identificados 136 processos judiciais do medicamento Zolgensma para tratamento da AME no âmbito do Ministério da Saúde, dos quais 113 (83%) foram favoráveis aos beneficiários das ações judiciais. Características demográficas (sexo e idade), clínicas (subtipos da AME, uso de suporte ventilatório ou nutricional) e do processo judicial (tipo de serviço advocatício) não foram associadas com o deferimento das ações judiciais. Apenas o uso prévio de medicamento para AME (nusinersena ou ridisplam) foi associado com o indeferimento dos processos judiciais. A AME I foi o subtipo mais frequente nos processos judiciais, totalizando 118 processos (86,7%) do total identificado no período sob investigação (Tabela 1).

**Tabela 1:** Informações das demandas judiciais do medicamento Zolgensma no âmbito do Ministério da Saúde entre janeiro de 2019 e setembro de 2022, Brasil.

Característica	Demanda judicial deferida^a^		Demanda judicial indeferida		valor de p
	n° demandantes	%	n° demandantes	%	
Sexo					0,590
Feminino	52	46	12	52,2	
Masculino	61	54	11	47,8	
Idade do paciente no peticionamento					0,323
0–6 meses	31	27,4	4	17,4	
7–12 meses	33	29,2	9	39,1	
13–18 meses	27	23,9	5	21,7	
19–24 meses	19	16,8	3	13,0	
Igual ou acima de 25 meses	3	2,7	2	8,7	
Idade do paciente no cumprimento judicial^a^					
0–6 meses	10	9,2			
7–12 meses	16	14,7			
13–18 meses	33	30,3			
19–24 meses	32	29,4			
Igual ou acima de 25 meses	18	16,5			
Tipo de AME					0,438
I	99	87,6	19	82,6	
II	13	11,5	3	13	
III	1	0,9	1	4,3	
Tipo de representação					0,161
Privada	110	97,3	21	91,3	
Pública	3	2,7	2	8,7	
Uso de outros medicamentos^b^					0,025
Sim	45	39,8	15	65,2	
Não	68	60,2	8	34,8	
Suporte ventilatório					0,701
Sim	54	47,8	12	52,2	
Não	59	52,2	11	47,8	
Suporte nutricional					0,974
Sim	34	30,1	7	30,4	
Não	79	69,9	16	69,6	

^a^ Dos 113 processos deferidos, quatro ainda não tinha a data de atendimento do cumprimento judicial. ^b^ Medicamentos para o manejo da AME: nusinersena ou risdiplam.

Ao analisar apenas os casos de deferimento das ações judiciais, 97,3% dos pacientes tinham idade de até dois anos quando do início do processo judicial, estando convergentes com as recomendações de bula do fabricante do medicamento. No entanto, esse percentual cai para 84,5% ao se analisar a idade do paciente quando do cumprimento da ação judicial (pagamento do medicamento). Levando em consideração o limite de até seis meses de idade para a obtenção do Zolgensma para o tratamento da AME estabelecido em protocolo clínico no Brasil pela Conitec e outras agências internacionais (CADTH e NICE), esses percentuais se reduzem a 27,4% e 9,2%, respectivamente (Tabela 1).

Ressalta-se que, dos 113 processos com ações deferidas, em apenas 109 deles havia informações do custo e da idade no cumprimento. Quatro processos deferidos ainda não tinham sido atendidos com a entrega do medicamento ou depósito judicial. No período do estudo, o Ministério da Saúde desembolsou R$ 944,8 milhões para atender 109 demandas judiciais do medicamento Zolgensma para tratamento da AME, com um gasto médio de R$ 8,67 milhões por paciente. Em 43 processos judiciais, foram solicitados recursos adicionais para pagamento de honorários médicos, hospitalizações e transporte dos pacientes ao local onde ocorreria a aplicação do medicamento, totalizando R$ 3,2 milhões no período investigado (Tabela 2).

**Tabela 2:** Gasto total despendido pelo Ministério da Saúde com demandas judiciais do medicamento Zolgensma por tipo de AME no período de janeiro de 2019 a setembro de 2022.

Tipo de AME	Custo com medicamento		Custos adicionais		Custo total (R$)
	Quantidade	Custo (R$)	Quantidade	Custo (R$)	
I	99^a^	833.386.445,27	37	2.950.690,29	836.337.135,56
II	13^b^	99.377.369,67	6	271.240,14	99.648.609,81
III	1	8.812.225,00	0	R$ 0,00	8.812.225,00
Custo total					944.797.970,37

^a^ Três ações judiciais deferidas ainda não haviam sido atendidas e, portanto, não foram considerados seus custos.

^b^ Uma ação judicial deferida ainda não havia sido atendida e, portanto, não foram considerados os seus custos.

A Tabela 3 relaciona os gastos da judicialização do medicamento Zolgensma com os critérios definidos em protocolos clínicos em diferentes países, incluindo o Brasil. Das 113 ações judiciais concedidas em favor dos pacientes, apenas seis (5,3%) atenderiam os critérios estabelecidos pela Conitec (crianças com até seis meses sem suporte ventilatório e nutricional). Dessa forma, R$ 891 milhões despedidos com ações judiciais não atenderiam os critérios estabelecidos em protocolo clínico no Brasil. Outro ponto que vale ressaltar é o dispêndio de R$ 146 milhões com fornecimento do Zolgensma para crianças com idade superior a dois anos, que está fora da recomendação contida na bula do medicamento, estabelecida pelo fabricante (Tabela 3).

**Tabela 3:** Quantidade e gasto incorrido pelo Ministério da Saúde para atender a demanda judicial do Zolgensma para tratamento da AME, estratificados por critérios clínicos adotados por agências internacionais de avaliação de tecnologias em saúde.

Grupo	AME tipo I		AME tipo II			AME tipo III
	n	Custo (R$)	n	Custo (R$)	n	Custo (R$)
Crianças até seis meses sem suporte ventilatório e nutricional	6	53.782.491,83	0	0,00	0	0,00
Crianças até seis meses com suporte ventilatório e nutricional	4	36.731.461,25	0	0,00	0	0,00
Crianças > seis meses e ≤ 24 meses com suporte ventilatório e nutricional	42	384.831.441,21	0	0,00	0	0,00
Crianças > seis meses e ≤ 24 meses sem suporte ventilatório e nutricional	30	247.183.951,57	9	76.249.872,02	0	0,00
Crianças > 24 meses	17^a^	113.807.789,70	4^b^	23.398.737,79	1	8.812.225,00
Total	99	836.337.135,56	13	99.648.609,81	1	8.812.225,00

^a^ Três ações judiciais deferidas ainda não haviam sido atendidas e, portanto, não foram considerados seus custos.

^b^ Uma ação judicial deferida ainda não havia sido atendida e, portanto, não foram considerados os seus custos.

## DISCUSSÃO

Durante o período analisado, o Ministério da Saúde desembolsou R$ 944,8 milhões para o cumprimento dos processos judiciais do medicamento Zolgensma para o tratamento da AME. A média anual foi de R$ 251,9 milhões para o atendimento de aproximadamente 29 pacientes, configurando-se na maior despesa com judicialização em âmbito federal. Excluindo-se os gastos com o Zolgensma para tratamento da AME, o Ministério da Saúde desembolsou R$ 802,6 milhões com o atendimento de outras ações judiciais em 2019, atendendo mais de 3.000 beneficiários^26^. A comparação torna-se mais díspar quando é considerado o gasto total com medicamentos no SUS, que totalizou R$ 10,29 bilhões no mesmo ano^27^, incluindo o atendimento de todas as doenças e agravos de saúde no país pela via das políticas de saúde, o que, em tese, representa o atendimento a mais de 200 milhões de habitantes. O gasto da judicialização do Zolgensma para AME representaria 2,45% do gasto total com medicamentos no SUS, incluindo gastos das três esferas administrativas (Ministério da Saúde, estados e municípios). Cabe ressaltar ainda que, do gasto total com a judicialização do Zolgensma para AME (R$ 944,8 milhões), apenas R$ 53,8 milhões (5,7%) atenderia aos critérios estabelecidos pela Conitec e R$ 798,7 milhões (84,5%) em relação à bula do fabricante do medicamento. Contudo, considerando que a própria bula não garante eficácia e segurança em pacientes com paralisia total dos membros e dependência permanente de ventilação, este gasto pode ser maior, pois não se sabe se o uso de suporte ventilatório informado era permanente.

O valor desembolsado também variou em termos de forma de pagamento. De 109 processos atendidos, em apenas 18 deles a compra do medicamento ocorreu diretamente pelo Ministério da Saúde, com o valor unitário do *kit* de R$ 5.722.712,79. Para os casos em que houve depósito para compra direta pelo beneficiário ou responsável pela ação, a média do valor do medicamento foi de R$ 9.215.849,51 (variando de R$ 4.179.008,39 a R$ 12.105.487,50). Em 23 processos de depósito, o *kit* do medicamento ultrapassou R$ 11.000.000,00, chegando a R$ 12.105.487,50. O valor pago via depósito é superior ao valor regulado pela Câmara de Regulação do Mercado de Medicamentos (CMED), estabelecido em R$ 6,5 milhões^15^.

Os tipos de AME deste estudo encontram semelhanças com os dados publicados a respeito da doença. A maior parte dos processos deferidos correspondem a pacientes portadores de AME tipo I, justamente por ser o subtipo mais grave^28^, que demanda maiores cuidados e que tem maior prevalência no Brasil^29^.

A ausência de tratamento medicamentoso previamente contribuiu para a decisão de conceder o Zolgensma nos processos judiciais no contexto do Ministério da Saúde. Esse padrão está em consonância com as evidências disponíveis, visto que a exposição a tratamentos prévios foi um dos critérios de exclusão nos ensaios clínicos^30,31^. O mesmo não ocorreu em termos de idade do início do tratamento (até seis meses) e uso de suporte ventilatório e nutricional. Os ensaios clínicos disponíveis incluíram apenas pacientes com idade até seis meses e portadores de duas cópias do gene SMN2; entre os critérios de exclusão, estava o uso de suporte ventilatório invasivo (traqueotomia) ou suporte ventilatório não invasivo com média ≥ 6 horas diárias, bem como pacientes que apresentavam problemas de deglutição (sinais de aspiração/incapacidade de tolerar líquido)^32^. A idade das crianças é um fator bastante relevante para o tratamento, pois, uma vez instalado os danos motores, não há como revertê-los^35^.

No *Clinical Trials*, existem 13 estudos do medicamento Zolgensma para o tratamento da AME (busca feita em 1^o^ de outubro de 2023). Contudo, apenas cinco possuem resultados publicados (NCT03837184, NCT03306277, NCT03461289, NCT03505099, NCT03381729). Em todos os estudos com resultados, as crianças possuíam idade igual ou inferior a seis meses, apresentavam até duas cópias do gene SMN2, não estavam em suporte ventilatório invasivo ou não invasivo por mais de ≥6 horas diárias e em suporte nutricional^30,31,33,34,36^. Na maioria dos processos judiciais deferidos, ao considerar a data de peticionamento, 72,6% das crianças já possuíam idade superior a seis meses. Ainda, é válido mencionar que a própria bula do medicamento indica o tratamento para pacientes pediátricos até dois anos de idade^8^. Ao total, 16,5% das crianças aplicaram o medicamento acima de dois anos de idade.

Outras agências internacionais também restringiram o uso do Zolgensma para tratamento da AME a determinadas condições clínicas e idade do início do tratamento. A agência canadense e o NICE da Inglaterra limitaram o uso do medicamento a pacientes de até seis meses de idade, com AME tipo I, sem necessidade de suporte alimentar ou suporte ventilatório (invasivo ou não invasivo)^17,18^. Essas recomendações são semelhantes a adotadas no Brasil, recomendadas pela Conitec. Na Escócia, o medicamento foi aprovado para reembolso apenas para pacientes menores de um ano que estiverem em fase pré-sintomática, ou seja, para aqueles que ainda não apresentaram sintomas da doença^17^. O *Institute for Clinical and Economical Review* (ICER) avaliou o medicamento e comparou a custo-efetividade com nusinersena, concluindo que o Zolgensma é mais custo-efetivo se aplicado na fase pré-sintomática de pacientes sem sintomas ou menores que dois/três meses^37^.

A grande quantidade de ações judiciais para obtenção do Zolgensma fora dos parâmetros indicados pela Conitec (idade de até seis meses e ausência de suporte ventilatório e nutricional)^13^pode estar relacionada à dificuldade de obtenção de diagnóstico precoce da AME no Brasil. Por ser uma desordem de baixa incidência e que evolui progressivamente, o diagnóstico da AME pode se tornar um desafio, visto que sinais da AME podem ser confundidos com outras neuropatologias^38^. Além disso, há dificuldades para conhecer os sinais clínicos, a falta de especialistas e a ausência de teste genético para confirmação do diagnóstico. A pesquisa “Retrato da AME no Brasil” mostrou que apenas 1% dos pacientes havia sido diagnosticado antes do nascimento e 11%, até um ano de idade. Ademais, cerca de 33% dos indivíduos relatam que o diagnóstico só foi possível após consultar cinco médicos ou mais^39^. Um estudo em grupos de pacientes da Europa, Austrália, Estados Unidos e Ásia comparou a idade de aparecimento dos primeiros sintomas da AME e a idade de confirmação do diagnóstico. Os resultados mostraram que atrasos de diagnóstico podem levar meses ou anos^40^.

Mesmo com os benefícios apresentados pelos estudos de eficácia do medicamento Zolgensma para a população com até seis meses de idade, sem algumas comorbidades definidas (como o uso de suporte ventilatório), ainda existe uma discussão sobre a relevância clínica dos desfechos publicados. Até o momento, os estudos mostram que o marco motor atingido pelos pacientes foi o alcance de posição independente por 30 segundos aos 18 meses de idade. Inclusive, alguns marcos foram redefinidos ao longo dos estudos, o que diminui a confiabilidade da análise dos dados^16^. Por se tratar de um tratamento novo, muitos estudos ainda estão em andamento e não há dados publicados sobre os reais benefícios com a terapia a longo prazo.

### Fortalezas e Limitações do Estudo

Este estudo contribuiu com a sumarização de dados administrativos sobre os processos judiciais contra o Ministério da Saúde para a obtenção do medicamento Zolgensma para AME. O desenho censitário em âmbito federal permitiu identificar os custos e a convergência do deferimento judicial aos critérios clínicos estabelecidos por agências internacionais de avaliação de tecnologias em saúde, incluindo a Conitec.

Algumas limitações do estudo precisam ser mencionadas. Primeiro, grande parte do período analisado ocorreu durante a pandemia de Covid-19, o que pode ter reduzido a procura por peticionamentos relativos à AME e aumentado as demandas relativas à Covid-19. Segundo, a ausência de informações mais detalhadas sobre o uso de suporte ventilatório nos documentos médicos. Acredita-se que o número de crianças em suporte ventilatório pode ser maior, pois, considerando a morosidade em realizar o cumprimento da ação judicial deferida, muitas crianças podem ter necessitado de maiores cuidados devido à progressão da doença. Somente um estudo mais detalhado desta coorte poderá conhecer o perfil dos pacientes durante a aplicação do medicamento e fornecer as respostas quanto aos benefícios do tratamento com o Zolgensma. Por fim, ressalta-se que não foi foco deste estudo avaliar se estão corretos os critérios usados pelas agências de avaliação de tecnologias em saúde, incluindo a Conitec, sobre a incorporação do Zolgensma nos sistemas de saúde. Assim, recomendam-se pesquisas futuras nesta direção.

### Implicações dos resultados para as políticas de saúde

Este estudo corrobora para a importância do diagnóstico precoce da AME e, consequentemente, sua distribuição no SUS, visto que mais da metade das crianças que judicializaram o medicamento estava fora dos parâmetros indicados pela Conitec. Além disso, verifica-se que a aplicação do medicamento ocorreu em crianças com perfil distinto ao indicado pelas evidências da literatura (estudos publicados e a própria bula do fabricante) e dos protocolos de recomendação elaborados por agências de avaliação de tecnologias em saúde.

Por ser uma condição de saúde de baixa incidência e que evolui progressivamente, o diagnóstico da AME pode se tornar um desafio e levar meses ou anos, devido à similaridade dos sintomas iniciais com outras neuropatologias, à falta de especialistas e à ausência de teste genético para confirmação do diagnóstico. Estes fatores podem implicar, por exemplo, o acesso do medicamento pelo SUS, conforme os critérios recomendados pela Conitec. As políticas públicas de saúde precisam fomentar o diagnóstico precoce da AME.

Destaca-se também a importância dos Núcleos de Apoio Técnico do Judiciário (NAT-JUS) na elaboração de pareceres fundamentados na medicina baseada em evidências e do Fórum de Saúde do Conselho Nacional de Justiça (CNJ) no monitoramento das demandas em saúde. A jurisprudência necessita deste apoio técnico para a realização de uma análise minuciosa do caso concreto, sendo primordial adotar a medicina baseada em evidências para a tomada de decisão. Os custos crescentes em saúde, o reconhecimento da limitação dos recursos, a necessidade de garantir os direitos constitucionais e a crescente intervenção do Poder Judiciário fazem com que a Avaliação de Tecnologias em Saúde (ATS) seja peça fundamental no processo de decisão.

Deve-se ampliar a discussão sobre a forma de cumprimento da ação judicial, visto que o pagamento direto pelo Ministério da Saúde obteve menores custos na aquisição do medicamento comparado ao depósito judicial. Obter-se-ia uma economia R$ 332 milhões no período investigado caso os 95 processos judiciais cumpridos pelo meio de depósito judicial fossem feitos por meio de pagamento direto.

Devido às incertezas sobre os efeitos do medicamento a longo prazo e às preocupações com a sustentabilidade orçamentária do SUS, ressalta-se a importância da implementação de acordos de compartilhamento de risco para medicamentos de alto custo utilizados em doenças raras, até mesmo para o cenário judicial. É importante que o judiciário tenha visão técnica adequada na análise das postulações e evitem proferir decisões contrárias ao sistema de saúde e à ciência.
